# On the Prognostic Implication of Delays in the Definitive Treatment of Uveal Melanoma

**DOI:** 10.3390/cancers16223834

**Published:** 2024-11-14

**Authors:** Gustav Stålhammar, Salvatore Grisanti, Paul T. Finger

**Affiliations:** 1 Ocular Oncology Service, St. Erik Ophthalmic Pathology Laboratory, St. Erik Eye Hospital, 171 64 Solna, Sweden; 2 Department of Clinical Neuroscience, Division of Eye and Vision, Karolinska Institute, 171 77 Stockholm, Sweden; 3 Department of Ophthalmology, University Medical Center Schleswig-Holstein Lubeck, University of Lübeck, 23538 Luebeck, Germany; 4 Department of Ocular Tumor, Orbital Disease, and Ophthalmic Radiation Therapy, The New York Eye Cancer Center, New York City, NY 10065, USA

**Keywords:** uveal melanoma, choroidal melanoma, delayed treatment, recurrence, metastases

## Abstract

**Background:** Recent studies suggest that delays in the definitive treatment of uveal melanoma may increase the risk of metastatic disease. This topic has been the subject of considerable debate. **Methods:** In this study, we combine and contrast medical evidence from several recent publications seeking to clarify the association between treatment delays and prognosis. **Results:** Emerging evidence indicates that metastatic seeding may continue until the primary tumor is effectively treated. Metastases that arise later in the disease course may carry additional genetic aberrations, enhancing their capacity to establish fatal macrometastases. Importantly, previous reports of shared mutations between primary tumors and metastases should not be interpreted as evidence that all metastases are seeded early. On the contrary, some tumors acquire additional driver mutations in the later stages, which are subsequently shared between the primary tumors and metastases. The increased risk of metastasis in patients with local tumor recurrence further highlights the importance of timely treatment. Additionally, new data on circulating tumor cells and treatment timing challenge the traditional practice of observing small melanomas. **Conclusions:** Observation is still warranted for indeterminate lesions to confirm malignancy. However, once a melanoma diagnosis has been established, further observation is harmful, and treatment should be administered as soon as reasonably possible.

## Simple Summary

Delays in treating uveal melanoma, a type of eye cancer, may increase the risk of the cancer spreading to other parts of the body. Traditionally, small eye tumors were often observed for growth before deciding on treatment, but new evidence suggests that this approach might allow the cancer to worsen. Our research explores the relationship between treatment delays and the risk of cancer spreading, showing that prompt treatment can reduce this risk. We also review the potential side effects of current treatments, such as radiation therapy, and highlight new strategies, like using medications to protect vision during treatment. The findings of this study may encourage eye specialists to adopt more timely and personalized treatment approaches, ultimately improving survival and quality of life for patients with uveal melanoma.

## 1. Introduction

Historically, observation for growth has been an accepted management approach for small choroidal melanomas [1]. Additional reasons that delays may occur even for larger tumors include patient hesitancy to undergo treatments that may compromise vision, comorbidities preventing general anesthesia, and limited access to healthcare resources, such as ocular oncologists and operating theaters (as further discussed in the Healthcare Restrictions Section), as well as the belief that delays in treatment do not impact the prognosis.

These practices and beliefs have been supported by a substantial body of literature suggesting that tumor cells disseminate early in the disease course. Studies have reported the early seeding of micrometastases to distant organs and shared genetic mutations between metastases and primary tumors, leading to the conclusion that treatment delays do not significantly affect survival outcomes [2,3,4,5]. Notably, a 1993 study by Augsburger comparing prompt versus delayed treatment found no significant difference in survival, reinforcing the perception that treatment timing is less critical [6].

However, this interpretation warrants reevaluation. For example, previous reports on shared mutations between primary tumors and metastases must not be misinterpreted as evidence that the metastases that eventually grow large and lead to patient death are always seeded very early in the life of a primary tumor. On the contrary, Shain and colleagues observed that while “metastatic dissemination occurred early during the development of the primary tumor,” they also found that some tumors acquired additional driver mutations in the branches of phylogenetic trees shared between the later stages of primary tumors and metastases [2]. These mutations included CDKN2A, the loss of chromosome 3 (encompassing PBRM1), the gain of 8q, and the gain of 1p. Judging by supplementary data from Shain’s study, approximately 5 out of 35 (14%) patients had metastases originating from the later branching parts of the phylogenetic trees (cases A11, A16, A50, A59, and A60). Two of these patients (A50 and A60) had small, potentially asymptomatic tumors, making earlier detection less likely. This suggests two things: first, that if effective primary tumor treatment, such as enucleation, had been performed in the three remaining cases with larger tumors before these additional genetic aberrations developed, the analyzed metastases might have been prevented; and second, that if tumor cells had already been seeded from older parts of these three tumors and managed to establish growing macrometastases, they would likely have carried fewer driver mutations, potentially leading to slower growth.

Given this theoretical maximal effect of timely primary tumor treatment—where up to 9% (3 out of 35, or 1 in 12) of metastases might be prevented or deferred if the primary tumor is treated before additional driver mutations arise (in practice, likely fewer)—it is unsurprising that studies of small cohorts, such as the one by Augsburger (*n* = 34 with delayed treatment, of which 7 died from metastatic uveal melanoma), fail to show a significant prognostic implication of treatment delays.

**Figure 1:**
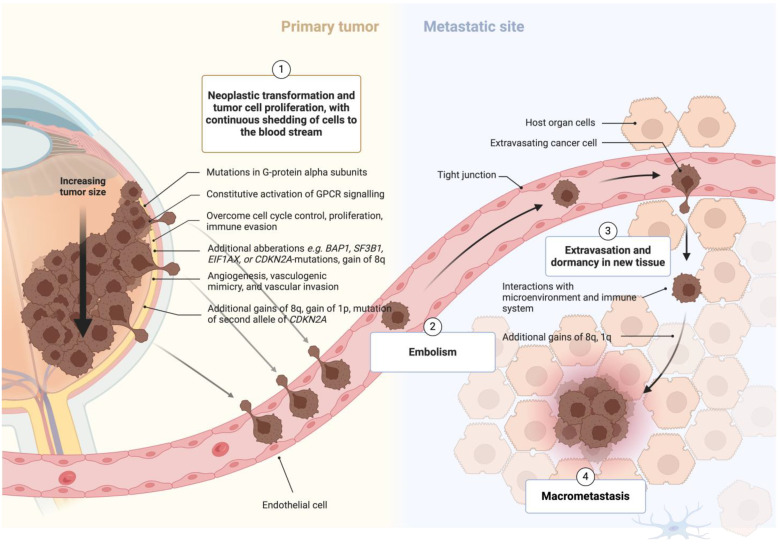
Schematic representation of the pathway from tumor cell transformation to metastasis (Created with https://www.biorender.com/).

Recent research further underscores the potential prognostic implications of delaying treatment in uveal melanoma. Grisanti’s work on circulating tumor cells demonstrated that even small uveal melanomas continually release cancer cells into the bloodstream. [7] While each circulating tumor cell represents a potential for metastasis, the complex process of metastatic spread involves more than just the presence of cancer cells. It requires the tumor embolus to survive, adhere to distant tissues, invade, undergo angiogenesis, and proliferate—all while evading the host’s immune system (Figure 1).

Stålhammar’s recent study demonstrated that delays between uveal melanoma diagnosis and treatment are indeed associated with an increased risk of metastatic death in a large cohort of 1145 patients [8]. Although the study did not include genetic data on the tumors, which is a limitation, it is unlikely that patients who experienced delays of more than 30 days had a higher proportion of aggressive genetic traits at baseline by chance, especially since these patients presented with smaller tumors and more frequently exhibited BAP-1 protein loss. A more plausible explanation for their worse prognosis is that the tumors continued to grow during the delay, resulting in a more advanced stage at the time of treatment. This is further supported by data from 12 cases where the tumor volume was measured at both diagnosis and treatment, showing that tumors had grown during the delay. The mean tumor volume increased from 355 mm^3^ to 769 mm^3^, corresponding to a doubling time of approximately 368 days.

This doubling time is consistent with findings from a recent systematic review and meta-analysis, which showed that uveal melanomas double in volume approximately every 360 days, with doubling times of 717, 421, and 307 days for small, medium, and large melanomas, respectively, and 6392 days for growing nevi [9]. A mixed effects model estimated that for every month a small, medium, or large melanoma remains untreated, the 10-year incidence of metastatic death increases by 0.3, 1.8, and 4.0 percentage points, respectively. Similar results were obtained using two independent survival data sources, suggesting that choroidal melanoma growth follows a super-exponential curve, with larger tumors exhibiting shorter doubling times, and delays in their treatment having a more severe impact on patient survival.

It is noteworthy that the 717-day doubling time for small melanomas is considerably shorter than the 4.3% growth rate observed for 24 uveal melanomas in another study by Damato and colleagues, which corresponds to a volume doubling time of approximately 2008 days, assuming a semi-spheroidal shape and similar growth in thickness and diameter [10]. This discrepancy likely explains the differences in conclusions between these studies. Damato et al. reported a worst-case scenario involving an “exceptionally fast” annual growth rate of 40%, corresponding to a doubling time of 752 days. However, as demonstrated in our meta-analysis, this may represent a rather slow growth rate, and even this scenario aligns with a metastatic death risk increase of 1.3% per month for a 40-year-old woman with a 10 mm diameter, monosomy 3 melanoma. In fact, a tumor with a yearly exponential growth rate of 74% was excluded “because of extremely rapid growth,” although this rate is close to the average growth rate found in our meta-analysis. In other words, this independent analysis, using separate datasets, reaches similar conclusions regarding the prognostic implications of treatment delays when accounting for differences in growth rates.

## 2. Clinical Management

The practice of observing small melanomas has become largely obsolete. However, it is crucial not to rush into treating indeterminate lesions, as observation for growth may be necessary to confirm the diagnosis of melanoma. In some cases, such as patients with a short life expectancy, the benefits of treatment are limited, and observation may be appropriate. But for most patients, once a melanoma diagnosis has been established, further observation becomes harmful, and treatment should be administered as soon as reasonably possible.

Biopsies are often used to confirm the diagnosis of melanoma and differentiate it from choroidal nevi or other similar lesions. Biopsies also help assess the tumor’s aggressiveness, which in turn may guide decisions regarding the necessity and urgency of treatment. However, in our experience, the usefulness of biopsies in determining if treatment is indicated is limited. Lesions that are very small and have no risk factors for growth (such as orange pigment, subretinal fluid, ultrasound hollowness, etc.) are rarely biopsied, and lesions with such risk factors very rarely exhibit purely benign histological characteristics. If a biopsy reveals a spindle A cell melanoma with favorable prognostic markers, such as disomy 3 or Gene Expression Class 1, will we withhold treatment? Despite the relatively low risk of metastatic death in such cases compared to other uveal melanomas, the risk remains significant when compared to other cancers—cancers we would not hesitate to treat.

Another factor influencing the decision to observe rather than treat is experience with ophthalmic radiation therapy. Although plaque brachytherapy is designed to spare both the eye and vision, radiation therapy for macular and peri-macular choroidal melanomas is well known to cause vision loss, with the radiation dose to the fovea strongly predictive of maculopathy [11,12]. However, since the introduction of intravitreal anti-vascular endothelial growth factor (anti-VEGF) therapy in 2006, multiple studies have demonstrated that vision loss from radiation maculopathy can often be delayed or even prevented [13,14].

If a treatment existed that did not pose a risk to vision, it is reasonable to assume that nearly all patients with suspicious choroidal melanocytic tumors would be treated immediately rather than observed.

## 3. Healthcare Restrictions

Reasons for delays in treatment may include hesitancy from either attending ophthalmologists or patients themselves, concerns over vision impairment, sociodemographic factors, and comorbidities [8,15,16]. Furthermore, restrictive referral patterns exist within all healthcare systems, with the simplest being a lack of available subspecialists or resource-based restrictions (patient, insurance, non-governmental, and governmental). This is a complex subject beyond the scope of this editorial but let us consider one example: the availability of radiation therapy. Cost drives these choices, ranging from a lack of eye- and vision-sparing therapy in low-resource countries to pressure to use reusable plaques. The latter reduces costs related to medical physics personnel and disposable seed sources. The most expensive alternative can also delay treatment, as patients must often travel to other countries for proton beam irradiation. However, all these treatments can cause dose-dependent irreversible radiation maculopathy, optic neuropathy, keratitis sicca, or neovascular glaucoma, leading to treatment hesitancy and recommendations for observation [17]. An additional consideration, particularly relevant for ophthalmologists in non-tertiary centers, is the importance of accurate referral to specialized centers with expertise in ocular oncology. Ensuring that only high-risk or concerning choroidal lesions are referred allows subspecialists to focus on cases requiring prompt intervention, minimizing diagnostic delays and optimizing treatment planning. Establishing streamlined pathways for accurate referral may help ensure that patients receive timely, effective care without overwhelming specialist resources.

## 4. Local Treatment Failure

Local recurrence has been described anatomically as apical or marginal. While the latter may be associated with the geographic misplacement of the radiation (plaque or proton), extrascleral extension, or eye movements, the central failure of local control is more likely linked to an insufficient dose or tumor-specific, genetic, or hypoxia-related factors [18]. In either scenario, a population of tumor cells have evidently survived the initial treatment. The longer these cells remain viable and proliferative, the more extended the period during which they can release tumor cells into the bloodstream. Importantly, the sustained viability of this tumor cell population over time should inherently increase the likelihood that the tumor will develop more aggressive characteristics. Therefore, local tumor recurrences should be considered another form of delay in definitive treatment.

Studies have revealed an increased risk of metastatic uveal melanoma for patients suffering locally recurrent disease (Table 1). For example, an international multicenter registry study of 3809 patients found that 152 (4.0%) developed locally recurrent disease and suffered a significantly increased metastatic rate (Hazard Ratio of 6.28 (95% CI, 4.4–8.9; *p* < 0.001), with the local recurrence treated as a time-varying covariate [19]. This risk was greater than, but consistent with, the prior literature published by the Collaborative Ocular Melanoma Study (COMS), Vrabec, Caujoulle, and Stålhammar [18,20,21]. Nonetheless, it has been debated whether the tumor recurrence is the cause of metastatic disease in itself or merely an indicator of increased metastatic potential from the onset [19].

Stålhammar compared patients treated with 15 mm vs. 20 mm brachytherapy plaques without the intraoperative verification of the plaque position [22]. Patients treated with the 15 mm plaque had significantly smaller tumors, smaller margins of plaque–tumor overlap, and a significantly higher competing risk incidence of local recurrence—which in turn was associated with metastatic death in a Cox regression with the tumor recurrence as a time-varying covariate. If we were to interpret local recurrence solely as an indicator of a tumor’s inherent metastatic potential, it would suggest that tumors treated with the 15 mm plaques inherently possessed more aggressive characteristics compared to the significantly larger tumors treated with the 20 mm plaques. However, this perspective leads to a paradox: despite their supposed aggressiveness, the smaller tumors treated with 15 mm plaques did not demonstrate a higher incidence of metastatic death. The logical interpretation of these findings is that local tumor recurrence itself contributes to an increased metastatic risk, rather than merely signaling an intrinsic propensity for metastasis from the disease onset. This view is corroborated by a recent study by Bagger and colleagues, who found that the tumors that recurred after plaque brachytherapy were not more likely to have an abnormal chromosome 3 and 8q status at the baseline [23].

## 5. Conclusions

There is mounting evidence that delays in or the failure of the local control of choroidal melanoma are associated with an increased risk of metastatic death. The magnitude of this effect warrants careful discussion, and each intervention must be weighed against its risks, potential benefits, side effects, and, notably, the risk of visual impairment. Many patients’ fatal metastases may have been established outside the eye well before the primary tumor was diagnosed and treated, meaning prompt treatment may only have an incremental effect on survival. However, as ocular oncologists, our commitment must be to explore every avenue to enhance patient outcomes, recognizing that even modest improvements in local treatment efficacy are crucial, and that the high mortality rate associated with uveal melanoma should not overshadow the importance of addressing incremental gains in survival. Our efforts should focus on distinguishing uveal melanomas from their benign counterparts and providing appropriate treatment at the earliest possible stage.

To address these challenges, early treatment following diagnosis is essential. Personalized medicine can help minimize the side effects of radiation therapy. For example, patients treated with custom-loaded plaques are more likely to preserve vision [24]. The American Brachytherapy Society consensus guidelines for plaque brachytherapy in uveal melanoma recommend calculating the radiation dose to the fovea and optic nerve to predict which patients are at risk for radiation vasculopathy-related vision impairment [25]. Should radiation maculopathy occur, intravitreal anti-VEGF therapy has been shown to prevent or delay vision loss [13,14].

Moreover, the multicenter, international PMCCC-centered Study of Ophthalmic Radiation Therapy Toxicity (SORTT) aims to identify source-specific radiation toxicity and efficacy. These personalized care initiatives empower eye cancer specialists to protect both the vision and the lives of our patients.

## Figures and Tables

**Table 1:** Studies of the relative risk of metastasis and death associated with local recurrence.

Authors	Treatment	Single/Multicenter	Median Follow Up Years	Number of Patients	Number with Local Recurrence	Percent Local Failure	Risk of Metastasis or Death with Local Recurrence
COMS [20]	Iodine-125 Plaque	Multicenter	5.6	650	57	10.3	Adjusted Risk Ratio: 1.5 (*p* = 0.08)
Vrabec [21]	Cobalt-60 Plaque	Single	4.9	445	70	15.7	5 yr Survival: 87 versus 58% with LR (*p* < 0.0001)
Caujoulle [18]	Proton	Single	5.1	1102	61	6.1	Overall survival at 10 years: 83.6 LR-free versus 43.1% with LR
Stålhammar [22]	Ruthenium-106	Single	10.8	1387	261	18.8	Hazard Ratio: 2.26 for LR (*p* < 0.001)
OOTF [19]	Varied	Multicenter	3.7	3217	152	4.7	Hazard Ratio: 6.3 for LR (*p* < 0.001)

COMS, Collaborative Ocular Melanoma Study; OOTF, Ophthalmic Oncology Task Force.

## Data Availability

No new data were created or analyzed in this study. Data sharing is not applicable to this article.
